# HIV-1 drug resistance and genetic transmission network among newly diagnosed people living with HIV/AIDS in Ningbo, China between 2018 and 2021

**DOI:** 10.1186/s12985-023-02193-x

**Published:** 2023-10-13

**Authors:** Hang Hong, Chunlan Tang, Yuhui Liu, Haibo Jiang, Ting Fang, Guozhang Xu

**Affiliations:** 1 School of Public health, Health Science Center, Ningbo University, Ningbo, Zhengjiang 315211 China; 2 Ningbo Center for Disease Control and Prevention, Ningbo, Zhengjiang 315010 China

**Keywords:** HIV/AIDS, Drug resistance, Genetic transmission network, Molecular epidemiology

## Abstract

**Background:**

As the HIV epidemic continues to grow, transmitted drug resistance(TDR) and determining relationship of HIV transmission are major barriers to reduce the risk of HIV transmissions.This study aimed to examine the molecular epidemiology and TDR and evaluated the transmission pattern among newly diagnosed people living with HIV/AIDS(PLWHA) in Ningbo city, which could contribute to the development of targeted precision interventions.

**Methods:**

Consecutive cross-sectional surveys were conducted in Ningbo City between January 2018 and December 2021. The HIV-1 pol gene region was amplified and sequenced for drug resistance and genetic transmission network analysis. TDR was determined using the Stanford University HIV Drug Resistance Database. Genetic transmission network was visualized using Cytoscape with the genetic distance threshold of 0.013.

**Results:**

A total of 1006 sequences were sequenced successfully, of which 61 (6.1%) showed evidence of TDR. The most common mutations were K103N (2.3%), E138A/G/Q (1.7%) and V179D/E (1.2%). 12 HIV-1 genotypes were identified, with CRF07_BC being the major genotype (43.3%, 332/767), followed by CRF01_AE (33.7%, 339/1006). 444 (44.1%) pol sequences formed 856 links within 120 transmission clusters in the network. An increasing trend in clustering rate between 2018 and 2021(*χ*^*2*^ = 9.546, *P =* 0.023) was observed. The odds of older age (≥ 60 years:OR = 2.038, 95%CI = 1.072 ~ 3.872, compared to < 25 years), HIV-1 genotypes (CRF07_BC: OR = 2.147, 95%CI = 1.582 ~ 2.914; CRF55_01B:OR = 2.217, 95%CI = 1.201 ~ 4.091, compared to CRF01_AE) were significantly related to clustering. Compared with CRF01_AE, CRF07_BC were prone to form larger clusters. The largest cluster with CRF07_BC was increased from 15 cases in 2018 to 83 cases in 2021.

**Conclusions:**

This study revealed distribution of HIV-1 genotypes, and genetic transmission network were diverse and complex in Ningbo city. The prevalence of TDR was moderate, and NVP and EFV were high-level NNRTI resistance. Individuals aged ≥ 60 years old were more easily detected in the networks and CRF07_BC were prone to form rapid growth and larger clusters. These date suggested that surveillance and comprehensive intervention should be designed for key rapid growth clusters to reduce the potential risk factors of HIV-1 transmission.

## Background

Despite substantial efforts to control human immunodeficiency virus-1 (HIV-1), acquired immunodeficiency syndrome (AIDS) is one of the most serious public health problem worldwide [1]. At the end of 2021, there were 38.4 million people living with HIV/AIDS(PLWHA), and 28.7 million people were accessing antiretroviral therapy(ART) [2]. World Health Organization(WHO) revised global recommendations that ART should be initiated in everyone living with HIV, regardless of CD4 + T lymphocytes counts in 2015 [3]. Early ART can suppress viral replication, reduce opportunistic infections, and significantly reduce morbidity and mortality among PLWHA [4–6]. Since HPTN 052 study published in interim form in 2011 [7], the strategy of treatment as prevention had been generalized highly effective in stopping reducing the risk of HIV transmissions globally [8, 9]. However, with the widespread use of ART, HIV drug resistance would lead to treatment failure in HIV infected patients, and even increase HIV transmission among individuals with risk behaviors and transmitted drug resistance(TDR) [10, 11].

As the HIV epidemic continues to spread, it is necessary to locate the source and relationship of HIV transmission [12, 13]. Genetic transmission network, which constructed based on the genetic information of people infected with HIV through gene distance between sequences, widely used in the study of early HIV cases detection, long-term monitoring of drug resistance and targeted precision intervention [11, 14–17]. Real-time genetic transmission network have been improved and further combined with social transmission network to judge new transmission events, identify high-risk spreaders and potential infected individuals and evaluate the effect of intervention measures [18–20]. Clustering analyses based on HIV drug resistance surveillance would rapidly detect and respond to emerging clusters of HIV infection to further reduce new transmissions, which is one pillar of the ending the HIV Epidemic plan in the United States [21].

Ningbo is an eastern coastal city of China, nearby Shanghai, with an area of 9365 km^2^ and a population of approximately 9.54 million people. We previously conducted an HIV tracing epidemiological survey among MSM in Ningbo City during 2018–2020 [22]. This study aimed to examine the molecular epidemiology and TDR of HIV-1 and evaluated the transmission pattern among newly diagnosed PLWHA in Ningbo city during 2018–2021, which could contribute to the development of targeted precision interventions.

## Methods

### Study participants

A cross-sectional survey was conducted in Ningbo City between January, 2018 and December, 2021. The inclusion criteria were as follows: (1) aged 18 years and above; (2) newly diagnosed HIV-1 cases; (3) had no received ART before enrollment; (4) agreed to participate in the survey and signed an informed consent. After providing written informed consent, blood samples were collected for CD4 + T lymphocytes counts, drug resistance and HIV sequencing.

### Laboratory tests

Blood specimens were sent to Ningbo CDC for below laboratory tests. CD4 + T lymphocytes counts determined in fresh whole blood by Flow cytometry (Becton Dickinson, NJ, USA). The remaining whole blood sample was centrifuged at 3000 rpm to produce blood plasma preserved in a -80℃ freezer. Viral RNA was extracted from blood plasma using Viral RNA Mini Kit (Tianlong, Suzhou, China) according to the manufacturer’s instructions. The obtained RNA samples were amplified using reverse transcription polymerase chain reaction (PCR) and nested PCR for the pol (HXB2: 2147–3462, encoding the protease gene and the first 300 codons of the reverse transcriptase gene) gene regions of HIV-1. After electrophoretic analysis, the amplified positive products were sent to Hangzhou Qingke Zixi Biotechnology Co. Ltd. for purification and gene sequencing.

### Sequence analysis

The sequences were assembled and adjusted with Sequencher v5.0 software(Genecodes, Ann Arbor, MI). The assembled sequences were aligned, edited, and analyzed with Bio-Edit 7.2 software(Genecodes, Ann Arbor, MI). International reference sequences were selected and downloaded from the HIV databases of the Los Alamos National Laboratory (http://hiv.lanl.gov). To identify the HIV-1 subtypes, the phylogenetic tree of Neighbor-joining was constructed using Mega 11.0 software. Bayesian Information Criterion (BIC) scores was performed to determine the evolutionary model. Considering the models with the lowest scores, the Kimura 2-parameter model with 1000 bootstrap replicates was determined to be the best fitting model [23]. Sequences with possible intersubtype recombination were analyzed using the Recombination Identification Program (RIP) tool (https://www.hiv.lanl.gov/content/sequence/RIP/RIP.html).

### Drug resistance analysis

The sequences were submitted to the Stanford University HIV Resistance Database (http://hivdb.stanford.edu/) to describe and interpret HIV-1 TDR. TDR level was classified according to the Stanford Penalty Score as high (60), intermediate (30–59), or low (15–29) to the following drugs: PI, Protease inhibitor; NRTI, Nucleoside reverse transcriptase inhibitor; NNRTI, Non-nucleoside reverse transcriptase inhibitor; INSTI, Integrase strand transfer inhibitors; NFV: Nelfinavir; ABC: Abacavir; AZT: Zidovudine; D4T: Stavudine; DDI: Didanosine; FTC: Emtricitabine; 3TC, Lamivudine; TDF: Tenofovir; DOR: Doravirine; EFV: Efavirenz; ETR: Etravirine; RPV: Rilpivirine. Drug resistance mutations were analyzed using the CRP tool (http://cpr.stanford.edu/cpr.cgi).

### Genetic transmission network analysis

The genetic transmission network was inferred based on the nucleotide genetic distance (GD) between HIV-1 pol sequences from each participant. Hyphy2.2.4 software was used to calculate the GD between pairings based on the TN93 model. The GD threshold that could identify the maximum number of clusters in the genetic network was chosen for analysis [24]. Transmission partner was defined that the GD between two sequences was below the distance threshold. Cytoscape 3.6.0 software was used to construct HIV molecular transmission network diagram based on the optimal distance threshold of different gene distances and the number of molecular clusters. The GD threshold of 0.013 was chosen to identified the maximum number of clusters in the genetic network. A node in the network represents a case or sequence. The connection between two nodes was an edge or link. The number of node connections was the degree, indicating its importance in the network. The higher the degree, the higher the inferred communication relationship with more people, the higher the communication risk.The nucleotide sequences were submitted to GenBank under the accession numbers OR521366-OR522371.

### Statistical analysis

Characteristics of all participants were described by categorical variables presented as absolute values and percentages. The demographic information and distribution of genotype were examined by chi-square tests. Univariate and multivariable forward stepwise logistic regression models were performed to examine risk factors associated with study participants within genetic transmission networks. The statistical significance was defined as *P* < 0.05. All statistical analyses were performed in SPSS (version 21.0, IBM, Armonk, NY, USA).

## Results

### Participant characteristics

A total of 1097 newly diagnosed PLWH were enrolled in this study, and we successfully sequenced and analyzed the samples collected from 1006 (91.7%) individuals. Among them, the median age was 40 years (interquartile range 28–54 years), ranging from 18 to 84 years. There were 86.3% (868/1006) and 13.7% (138/1006) males and females, respectively. Regarding marital status, 37.5% (377/1006) were single, 42.8% (431/1006) were married, and 19.7% (198/1006) were divorce or death. Most participants were middle school or less education level (61.8%), of Han ethnicity (95.8%), living non local (56.5%), heterosexual transmission (52.7%), having < 6 sexual partners (79.6%). The proportions of CD4 + T lymphocytes counts < 200 cells/uL, 200 ~ 499 cells/uL and ≥ 500 cells/uL were 37.7% (379/1006), 52.2% (525/1006) and 10.1% (102/1006), respectively (Table 1).

**Table 1:** Characteristics of study participants between three groups with different HIV-1 genotypes

Characteristics	All participants n (%)		HIV-1 genotypes														χ^2^		P value	
			CRF01_AE n (%)		CRF07_BC n (%)		CRF08_BC n (%)			C n (%)			CRF55_01B n (%)		Others* n (%)					
**Overall**	1006(100)		339(100)		409(100)		69(100)			54(100)			50(100)		85(100)					
**Gender**																				
Male	868(86.3)		298(87.9)		366(89.5)		50(72.5)			42(77.8)			47(94.0)		65(76.5)		25.799		< 0.001	
Female	138(13.7)		41(12.1)		43(10.5)		19(27.5)			12(22.2)			3(6.0)		20(23.5)					
**Age (years)**																				
< 25	139(13.8)		50(14.7)		63(15.4)		5(7.2)			3(5.6)			7(14.0)		11(12.9)		51.906		< 0.001	
25~	356(35.4)		130(38.3)		149(36.4)		14(20.3)			20(37.0)			25(50.0)		18(21.2)					
40~	354(35.2)		110(32.4)		139(34.0)		26(37.7)			24(44.4)			18(36.0)		37(43.5)					
≥ 60	157(15.6)		49(14.5)		58(14.2)		24(34.8)			7(13.0)			0(0)		19(22.4)					
**Marital status**																				
Single	377(37.5)		135(39.8)		166(40.6)		16(23.2)			16(29.6)			20(40.0)		24(28.2)		17.299		0.068	
Married	431(42.8)		139(41.0)		160(39.1)		35(50.7)			28(51.9)			24(48.0)		45(52.9)					
Divorce or death	198(19.7)		65(19.2)		83(20.3)		18(26.1)			10(18.5)			6(12.0)		16(18.8)					
**Education level**																				
Middle school or less	622(61.8)		208(61.4)		242(59.2)		57(82.6)			35(64.8)			22(44.0)		58(68.2)		22.299		< 0.001	
High school or above	384(38.2)		131(38.6)		167(40.8)		12(17.4)			19(35.2)			28(56.0)		27(31.8)					
**Nationality**																				
Han	964(95.8)		329(97.1)		392(95.8)		64(92.8)			51(94.4)			47(94.0)		81(95.3)		4.534		0.450	
Others	42(4.2)		10(2.9)		17(4.2)		5(7.2)			3(5.6)			3(6.0)		4(4.7)					
**Location**																				
Local	438(43.5)		153(45.1)		167(40.8)		36(52.2)			27(50.0)			19(38.0)		36(42.4)		5.253		0.386	
Non local	568(56.5)		186(54.9)		242(59.2)		33(47.8)			27(50.0)			31(62.0)		49(57.6)					
**Route of infection**																				
Heterosexual transmission	532(52.7)		185(54.6)		193(47.2)		56(81.2)			33(61.1)			17(34.0)		48(56.5)		42.681		< 0.001	
Homosexual transmission	470(46.7)		152(44.8)		215(52.6)		13(18.8)			20(37.0)			33(66.0)		37(43.5)					
Others	4(0.6)		2(0.6)		1(0.2)		0(0)			1(1.9)			0(0)		0(0)					
**Number of sexual partners in a life time**																				
<6	800(79.5)		270(79.6)		327(80.0)		52(75.4)			44(81.5)			37(74.0)		70(82.4)		4.921		0.896	
6 ~ 10	130(12.9)		45(13.3)		50(12.2)		9(13.0)			6(11.1)			10(20.0)		10(11.8)					
≥11	76(7.6)		24(7.1)		32(7.8)		8(11.6)			4(7.4)			3(6.0)		5(5.9)					
**CD4,cell/ul**																				
< 200	379(37.7)		147(43.4)		138(33.7)		25(36.2)			19(35.2)			14(28.0)		36(42.4)		19.400		0.035	
200~	525(52.2)		161(47.5)		236(57.7)		32(46.4)			30(55.6)			29(58.0)		37(43.5)					
≥ 500	102(10.1)		31(9.1)		35(8.6)		12(17.4)			5(9.3)			7(14.0)		12(14.1)					
**Transmitted drug resistance**																				
Sensitive	945(93.9)		313(92.3)		399(97.6)		57(82.6)			49(90.7)			47(94.0)		80(94.1)		25.686		< 0.001	
Resistance	61(6.1)		26(7.7)		10(2.4)		12(17.4)			5(9.3)			3(6.0)		5(5.9)					

*: B,URFs, CRF85_BC,CRF01_BC,CRF59_01B, CRF67_01B and CRF68_01B

### Drug resistance analysis

**Fig. 1:**
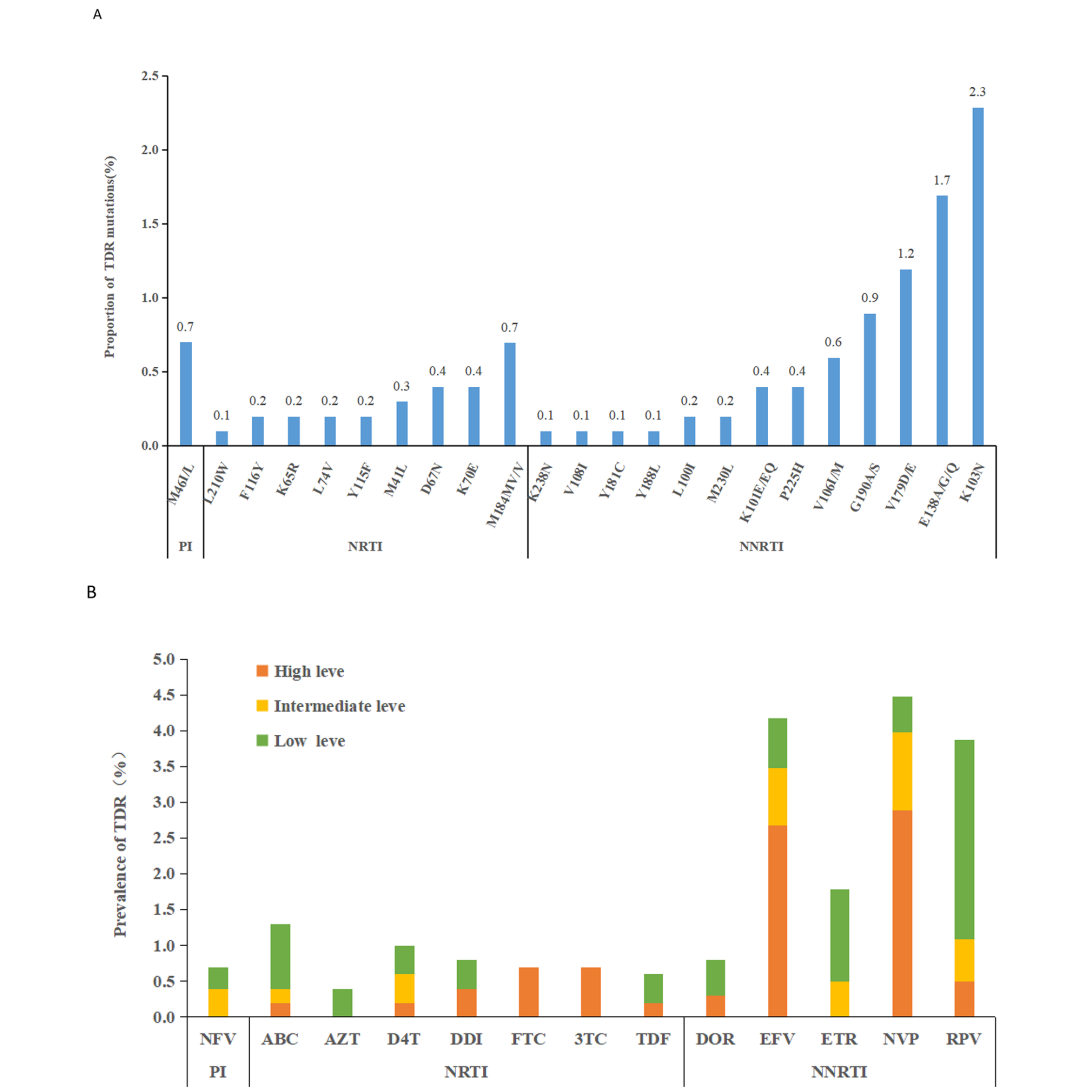
(**A**) Proportion of HIV-1 transmitted drug resistance (TDR) mutations to PIs, NRTIs, and NNRTIs. (**B**) Levels of HIV-1 TDR associated mutations to different ART drug among 1006 newly diagnosed HIV/AIDS patients. PI, Protease inhibitor;NRTI, Nucleoside reverse transcriptase inhibitor; NNRTI, Non-nucleoside reverse transcriptase inhibitor; NFV: Nelfinavir;ABC: Abacavir; AZT: Zidovudine; D4T: Stavudine; DDI: Didanosine; FTC: Emtricitabine;3TC, Lamivudine;TDF, Tenofovir; DOR,Doravirine; EFV,Efavirenz; ETR: Etravirine; RPV: Rilpivirine

The prevalence of TDR among newly diagnosed PLWH was 6.1% (61/1006). TDR-associated mutations to NNRTI, NRTI and PI were detected in 7.9% (79/1006), 2.7% (27/1006) and 0.7% (7/1006). No INSTI-related mutations were identified. As for the NNRTI resistance, NVP (2.9%) was the highest proportion of high-level resistance, followed by EFV (2.7%). Most exhibited a low-level resistance to RPV (2.8%) and ETR (1.3%). The most frequent mutations were K103N (2.3%), E138A/G/Q (1.7%) and V179D/E (1.2%). As for the NRTI resistance, FTC and 3TC presented the highest levels (0.7%), which was related to the mutation of M184MV/V (0.7%). As for the PI resistance, the HIV-1 strains only exhibited resistance to NFV (0.7%), and mutations were M46L/I(0.7%). The above results presented in Fig. 1.

### Genotype analysis

Among all participants, the most common genotype was CRF07_BC (40.7%, 409/1006), followed by CRF01_AE (33.7%, 339/1006). The other genotypes were CRF08_BC (6.9%, 69/1006), subtype C (5.4%, 54/1006), CRF55_01B (5.0%, 50/1006), subtype B (2.6%, 26/1006), unique recombinant forms (URFs) (2.6%, 26/1006), CRF85_BC (1.5%, 15/1006), CRF01_BC (0.6%, 6/1006), CRF59_01B (0.4%, 4/1006), CRF67_01B (0.4%, 4/1006) and CRF68_01B (0.4%, 4/1006). The URFs included 19 CRF01AE/C, four CRF01AE/B/C, two subtype B/C, one CRF01AE/B. As shown in Table 1, the distribution of genotype revealed significant differences according to gender (χ^2^ = 25.799, *P* < 0.001), age (χ^2^ = 51.906, *P* < 0.001), education level (χ^2^ = 22.299, *P* < 0.001), route of infection (χ^2^ = 42.681, *P* < 0.001), CD4 + T lymphocytes counts (χ^2^ = 19.400, *P* = 0.038), and TDR (χ^2^ = 25.686, *P* < 0.001).

### Genetic transmission network analysis

Overall, 444 (44.1%) pol sequences formed 856 links within 120 transmission clusters in the network. Comparing the sequences included to those not included in the transmission networks, we observed significant differences in age (*χ*^*2*^ = 4.778, *P =* 0.029), number of sexual partners in a life time (*χ*^*2*^ = 5.253, *P =* 0.022), HIV-1 genotypes (*χ*^*2*^ = 27.539, *P < 0.001*) and CD4 + T lymphocytes counts (*χ*^*2*^ = 6.581, *P =* 0.037). After adjustment for confounders, older age (*OR* = 2.038, *95%CI* = 1.072 ~ 3.872, *P* = 0.030; ≥60 vs. <25 years) was associated with higher adjusted odds of clustering. Additionally, compared with CRF01_AE, CRF07_BC (*OR* = 2.147, *95%CI* = 1.582 ~ 2.914, *P = 0.011*) and CRF55_01B (*OR* = 2.217, *95%CI* = 1.201 ~ 4.091, *P < 0.001*) were significantly related to clustering (Table 2).

**Table 2:** Factors associated with study participants within genetic transmission networks in Ningbo, China

Factors	Within genetic transmission networks n (%)	Univariate analysis		Multivariate analysis	
		*χ* ^*2*^	P value	*OR*(95%*CI*)	P value
**Gender**		2.231	0.135		
Male	375(84.5)			1.000	
Female	69(15.5)			1.252(0.815 ~ 1.923)	0.306
**Age (years)**		4.778	0.029		
< 25	55(12.4)			1.00	
25~	153(34.5)			1.248(0.814 ~ 1.914)	0.310
40~	152(34.2)			1.322(0.777 ~ 2.247)	0.303
≥ 60	84(18.9)			2.038(1.072 ~ 3.872)	0.030
**Marital status**		1.606	0.448		
Single	161(36.3)			1.000	
Married	200(45.0)			0.918(0.619 ~ 1.363)	0.673
Divorce or death	83(18.7)			0.784(0.502 ~ 1.226)	0.286
**Education level**		0.040	0.842		
Middle school or less	273(61.5)			1.000	
High school or above	171(38.5)			1.175(0.851 ~ 1.621)	0.328
**Nationality**		1.261	0.262		
Han	429(96.6)			1.000	
Others	15(3.4)			0.721(0.366 ~ 1.420)	0.344
**Location**		1.873	0.171		
Local	204(45.9)			1.000	
Non local	240(54.1)			0.950(0.712 ~ 1.267)	0.728
**Route of infection**		3.257	0.196		
Heterosexual transmission	248(55.9)			1.000	
Homosexual transmission	195(43.9)			0.888(0.642 ~ 1.228)	0.472
Others	1(0.1)			0.619(0.058 ~ 6.588)	0.691
**Number of sexual partners in a life time**		5.253	0.022		
<2	137(30.9)			1.000	
2~	307(69.1)			0.747(0.549 ~ 1.017)	0.064
**HIV-1 genotypes**		27.539	< 0.001		
CRF01_AE	112(25.2)			1.000	
CRF07_BC	211(47.5)			2.147(1.582 ~ 2.914)	< 0.001
CRF08_BC	30(6.8)			1.459(0.840 ~ 2.536)	0.180
C	25(5.6)			1.669(0.922 ~ 3.022)	0.091
CRF55_01B	25(5.6)			2.217(1.201 ~ 4.091)	0.011
Others^*^	41(9.2)			1.815(0.936 ~ 2.981)	0.069
**CD4,cell/ul**		6.581	0.037		
< 200	155(34.9)			1.000	
200~	250(56.3)			1.305(0.982 ~ 1.735)	0.067
≥ 500	39(8.8)			0.933(0.584 ~ 1.491)	0.773
**Transmitted drug resistance**		2.483	0.115		
Sensitive	423(95.3)			1.000	
Resistance	21(4.7)			1.384(0.784 ~ 2.444)	0.262

^*^: B,URFs, CRF85_BC,CRF01_BC,CRF59_01B, CRF67_01B and CRF68_01B

**Fig. 2:**
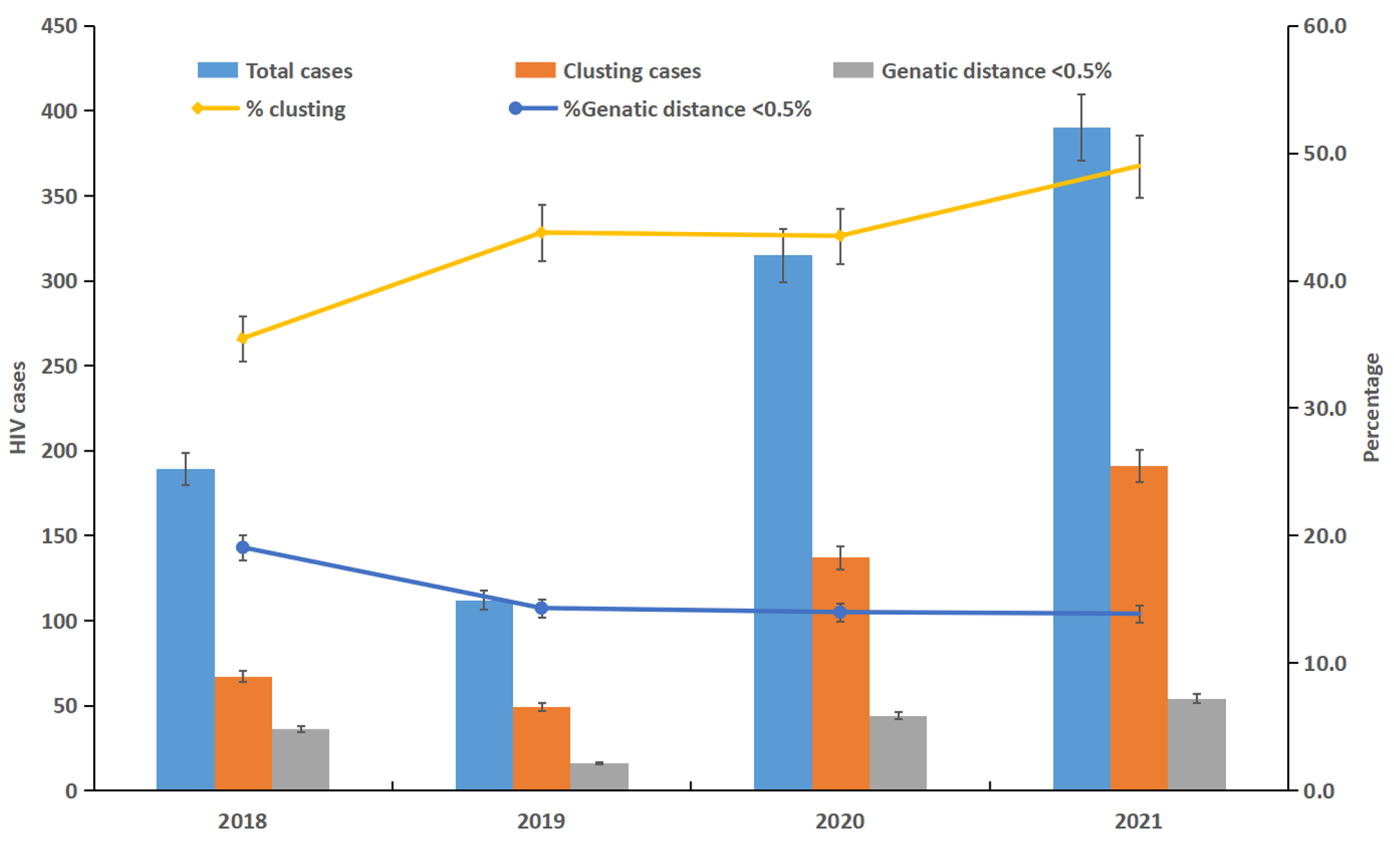
Enrolment and clustering rate in Ningbo, China between 2018 and 2021.Dispersion measures indicate 95% confidence intervals

As seen in the set of Fig. 2, the clustering rates were 35.4% (67/189), 43.8% (49/112), 43.5% (137/315), 49.0% (191/390) between 2018 and 2021, respectively. The proportions of links with GD < 0.005 were 19.0% (36/189), 14.3% (16/112), 14.0% (44/315), 13.8% (54/390), respectively. We observed an increasing trend in clustering rate between 2018 and 2021 (*χ*^*2*^ = 9.546, *P =* 0.023). The proportion of links with GD < 0.005 did not vary significantly during the four years (*χ*^*2*^ = 3.153, *P =* 0.369).

Of the 444 sequences evaluated, 154 (34.7%) had one link, and 290 (65.3%) had more than two links. There were 105 (87.5%) clusters with size < 5, 10 (8.3%) clusters with 5 ≤ size < 10, and five (4.2%) clusters with size ≥ 10. The five large clusters had 83, 21, 14, 10 and 10 individuals, respectively. The median degrees were 4, 20, 11, 7.5 and 9 respectively. The proportions of links with GDs < 0.005 were 19.0% (84/213), 42.9% (85/198) and 22.5% (16/71), 2.8% (1/36), 39.5% (17/43) respectively.

**Fig. 3:**
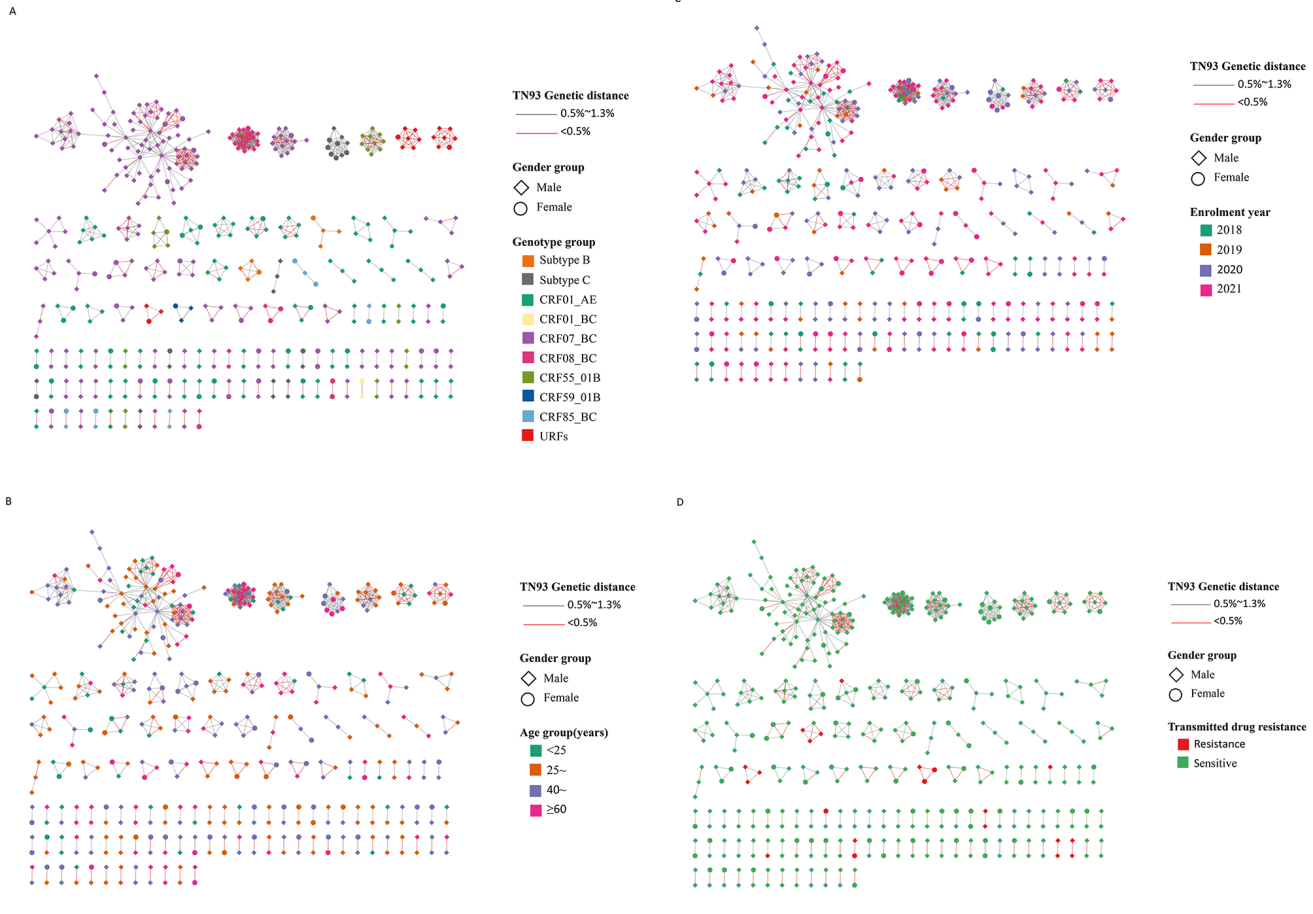
Genetic transmission network among newly diagnosed people living with HIV/AIDS in Ningbo, China between 2018 and 2021.Clusters are shown ordered by size in each panel. Links are coloured by genetic distance.Shapes of the nodes represent different gender.Colour of the nodes represent different participant characteristics,including genotype,age,enrolment year and transmitted drug resistance

As shown in Fig. 3, among 444 participants who were included in these networks, 375 (84.5%) were male and 69 (15.5%) were female. We analyzed these 120 clusters, 46 (38.3%) clusters were CRF07_BC, 42 (35.0%) were CRF01_AE, 8 (6.6%) were subtype C, 7 (5.8%) were CRF55_01B, 5 (4.2%) were CRF85_BC and CRF08_BC, 3 (2.5%) were URFs, 2 (1.6%) were subtype B, 1 (0.8%) were CRF01_BC and CRF59_01B. Of 211 CRF07_BC sequences, 137 (64.9%) were connected to at least one other sequence with genetics distance < 0.005. Compared with CRF01_AE, CRF07_BC were prone to form larger clusters. The largest cluster with CRF07_BC was increased from 15 cases in 2018 to 83 cases in 2021. Considering clusters with three or more nodes, the most frequent links included persons with 25–39 years (35.2%), followed 40–59 years (31.7%). The proportion of links with GD < 0.005 varied across age: 41.0%, 31.7%, 35.7% and 42.9% in persons < 25, 25–39, 40–59 and ≥ 60 years, respectively, and was significantly higher in persons ≥ 60 years compared to all other age teams (*χ*^*2*^ = 14.456, *P =* 0.002).Considering clusters included all the four years, there were two clusters with 83 and 21 nodes, respectively. The annual growth rate of participants who were included in these two clusters were 36.3% and 76.5%. We also observed that 34.4% (21/61) of TDR cases were included in the transmission. All these TDR cases were found in 11 clusters with size < 6. The possibility of TDR clustering was higher in gender being male (80.6%), infection route being heterosexual transmission (58.1%) and genotype being CRF01_AE (51.6%). Of 856 links in the genetic transmission networks, 13 (1.5%) links were TDR connected to TDR, 9 (1.1%) were TDR linked to No-TDR, and 834 (97.4%) were No-TDR connected to No-TDR.

## Discussion

The AIDS epidemic has a serious impact on health, economy and society of people all over the world. Meanwhile, TDR has become the focus of AIDS prevention and treatment. Furthermore, traditional epidemiological studies focus on the risk of individual HIV infection, with less consideration given to the impact of individual interactions on HIV transmission. Therefore, this study described TDR and genotype in newly diagnosed PLWHA in Ningbo city between 2018 and 2021, and further analyzed the HIV genetic network according to the GD. The results showed that the prevalence of TDR was 6.1% and 12 HIV-1 genotypes and 120 transmission clusters were identified, which revealed the complexity and diversity of the AIDS epidemic in Ningbo City.

Since China had adopted Free ART Program in 2003 and ‘Treat for all’ policy in 2016, free virus load test were implemented for all of the PLWHA receiving ART once per year [25].However, free viral load test is usually offered to patients until a year after ART to assess the effectiveness of treatment. Due to lack of resistance monitoring and testing before ART, the prevalence of TDR rapidly rising in recent years [26]. In our study, the prevalence of TDR was 6.1% and TDR-associated mutations to NNRTI was 7.9%, which was moderate (5–15%) according to the WHO definition [27]. It was higher than the prevalence determined in Sichuan [28], Guangdong [29] and Jiangsu [30] in China. In this study, we found that NVP and EFV showed high-level NNRTI resistance, with the most frequent mutations being K103N, E138A/G/Q and V179D/E. Additionally, in case of NRTI, the mutation M184MV/V can cause the highest level resistance to FTC and 3TC, which were consistent to the national study in China [26]. Moreover, 0.7% of subjects carried PI resistance related the mutation M46L/I which conferred TDR to NFV. Currently, 3TC, EFV and NVP are widely-used free drugs in the first-line ART regimens in China [31]. Long-term use of limited number of drugs facilitated the generation and spread of TDR. Therefore, the emergence of these mutations to 3TC, EFV and NVP should be closely monitored. In particular, drug-resistance testing prior to ART initiation should be performed to reduce spread of TDR. Furthermore, the introduction and development of longer-acting ART should be considered in China.

As is reported, HIV-1 genotype played an important role in HIV transmission and responses to ART [32]. Our study showed that the major epidemic genotypes were CRF07_BC and CRF01_AE, which was consistent to the prior study [33].And the distribution of genotype revealed significant differences according to gender, age, education level route of infection, CD4 + T lymphocytes counts and TDR. Currently, CRF08_BC transmission population has changed from intravenous drug users to heterosexuals in the highest risk groups in China [34]. CRF08_BC appeared to be a distinctive strain in Ningbo city. CRF08_BC was also the predominant genotype among individuals aged more than 40 years, middle school or less educational level, heterosexually transmitted individuals in our study. The prevalence of TDR was higher than other genotypes among individuals infected with CRF08_BC. This finding revealed urgent measures should be taken to interrupt the spread of CRF08_BC, particularly among elderly individuals. CRF55_01B, which was first detected in MSM, had quickly spread to heterosexuals, according to our results and earlier study [35]. Besides, CRF55_01B may have a higher transmission risk than CRF01_AE and CRF07_BC [36]. Thus, we should continue to concern the prevention and control of CRF55_01B.

We constructed HIV-1 genetic transmission network analysis to understand the transmission characteristic among PLWHA in Ningbo city. Our study showed that 444 individuals could be segregated into 120 transmission clusters. After adjusting for confounders, we observed that age and genotypes were significantly related to clustering. GD < 0.005 might be more appropriate for distinguishing rapidly growing clusters [37]. In this study, individuals aged over 60 years old were more likely to enter the transmission network and the proportions of links with GD < 0.005 were higher than other age teams, indicated that they increased the risk of local HIV transmission and should be monitored as a priority [38]. Moreover, compared with CRF01_AE, CRF07_BC were prone to form larger clusters and the largest cluster with CRF07_BC was grown rapid, which homosexual transmission was the major route of transmission. Furthermore, most of CRF07_BC sequences were connected to smaller GD. We found clustering rate was significantly increased between 2018 and 2021. It suggested that comprehensive intervention should be designed for key rapid growth clusters, especially for those with multiple links [39]. In addition, we identified that 34.4% of TDR cases were included in 11 clusters, mostly composed of heterosexuals. However, heterosexual contacts present small clusters, indicating a low level of forward transmission, but still represent a high proportion of TDR transmission in Ningbo city. These results suggested that local government should continue monitoring TDR transmission to conduct targeted interventions and control epidemics in a timely fashion [40].

### Limitation of this study

There are a few inherent limitations in this study. Firstly, the sample composition may not be representative all PLWAH in Ningbo city. Secondly, molecular transmission cluster only represented a group of highly associated infected individuals, and could not reflect direct transmission relationship. Thirdly, as integrase was not included in the amplified pol sequence, this study did not identify INSTI-related drug resistance mutations. Finally, with limited time and funds, it was based on sequence analysis of the pol gene, which may not holistically represent the epidemic.

## Conclusions

This study revealed distribution of HIV-1 genotypes and genetic transmission network were diverse and complex in Ningbo city. The prevalence of TDR was moderate, and NVP and EFV were high-level NNRTI resistance. Individuals aged ≥ 60 years old were more easily detected in the networks and CRF07_BC were prone to form rapid growth and larger clusters. These date suggested that surveillance and comprehensive intervention should be designed for key rapid growth clusters to reduce the potential risk factors of HIV-1 transmission.

## Data Availability

The datasets used and/or analyzed during this study is not publicly available, but may be available from the corresponding author upon reasonable request, and with permission from Ningbo Municipal Center for Disease Control and Prevention.

## Abbreviations

HIV: human immunodeficiency virus
AIDS: acquired immunodeficiency syndrome
PLWHA: people living with HIV/AIDS
ART: antiretroviral therapy
WHO: World Health Organization
TDR: transmitted drug resistance
PCR: polymerase chain reaction
RIP: Recombination Identification Program
PI: Protease inhibitor
NRTI: Nucleoside reverse transcriptase inhibitor
NNRTI: Non-nucleoside reverse transcriptase inhibitor
INSTI: Integrase strand transfer inhibitors
NFV: Nelfinavir
ABC: Abacavir
AZT: Zidovudine
D4T: Stavudine
DDI: Didanosine
FTC: Emtricitabine
3TC: Lamivudine
TDF: Tenofovir
DOR: Doravirine
EFV: Efavirenz
ETR: Etravirine
RPV: Rilpivirine
GD: genetic distance
URFs: unique recombinant forms
